# Varicella routine vaccination and the effects on varicella epidemiology – results from the Bavarian Varicella Surveillance Project (BaVariPro), 2006-2011

**DOI:** 10.1186/1471-2334-13-303

**Published:** 2013-07-02

**Authors:** Andrea Streng, Veit Grote, David Carr, Christine Hagemann, Johannes G Liese

**Affiliations:** 1 Department of Paediatrics, University of Würzburg, Josef-Schneider-Str. 2, D-97080, Würzburg Germany; 2 Department of Immunology and Infectiology, Children’s Hospital, University of Munich, Lindwurmstr 4, D-80337, Munich, Germany

**Keywords:** Varicella, Surveillance, Coverage, Vaccination, Hospitalisation, Paediatric, Incidence

## Abstract

**Background:**

In 2004, routine varicella vaccination was recommended in Germany for children 11-14 months of age with one dose, and since 2009, with a second dose at 15-23 months of age. The effects on varicella epidemiology were investigated.

**Methods:**

Data on varicella vaccinations, cases and complications were collected from annual parent surveys (2006-2011), monthly paediatric practice surveillance (Oct 2006 - Sep 2011; five varicella seasons) and paediatric hospital databases (2005-2009) in the area of Munich (about 238,000 paediatric inhabitants); annual incidences of cases and hospitalisations were estimated.

**Results:**

Varicella vaccination coverage (1^st^ dose) in children 18-36 months of age increased in two steps (38%, 51%, 53%, 53%, 66% and 68%); second-dose coverage reached 59% in the 2011 survey. A monthly mean of 82 (62%) practices participated; they applied a total of 50,059 first-dose and 40,541 second-dose varicella vaccinations, with preferential use of combined MMR-varicella vaccine after recommendation of two doses, and reported a total of 16,054 varicella cases <17 years of age. The mean number of cases decreased by 67% in two steps, from 6.6 (95%CI 6.1-7.0) per 1,000 patient contacts in season 2006/07 to 4.2 (95%CI 3.9-4.6) in 2007/08 and 4.0 (95%CI 3.6-4.3) in 2008/09, and further to 2.3 (95%CI 2.0-2.6) in 2009/10 and 2.2 (95%CI 1.9-2.5) in 2010/11. The decrease occurred in all paediatric age groups, indicating herd protection effects. Incidence of varicella was estimated as 78/1,000 children <17 years of age in 2006/07, and 19/1,000 in 2010/11. Vaccinated cases increased from 0.3 (95%0.2-0.3) per 1,000 patient contacts in 2006/07 to 0.4 (95%CI 0.3-0.5) until 2008/09 and decreased to 0.2 (95%CI 0.2-0.3) until 2010/11. The practices treated a total of 134 complicated cases, mainly with skin complications. The paediatric hospitals recorded a total of 178 varicella patients, including 40 (22.5%) with neurological complications and one (0.6%) fatality due to varicella pneumonia. Incidence of hospitalisations decreased from 7.6 per 100,000 children <17 years of age in 2005 to 4.3 in 2009, and from 21.0 to 4.7 in children <5 years of age.

**Conclusions:**

Overall, the results show increasing acceptance and a strong impact of the varicella vaccination program, even with still suboptimal vaccination coverage.

## Background

In the absence of a general vaccination programme, varicella (chickenpox), the primary infection with the varicella zoster virus (VZV), affects almost all children in the course of their childhood [1]. Varicella is usually mild in immunocompetent children, but occasionally there are severe complications and even fatalities [1-5]. To reduce general morbidity as well as the number of severe cases, several countries outside Europe (Australia, Canada, Costa Rica, Ecuador, Israel, New Zealand, Oman, Panama, Qatar, Saudi-Arabia, South Korea, Taiwan, the United Arab Emirates, Uruguay and USA) [1,6-8] as well as some European countries (Germany, Greece, Latvia, Luxembourg) [8-11] and regions (seven out of 21 in Italy [12], two out of 17 in Spain [13]) have introduced routine varicella vaccination during the last two decades.

The United States was the first country to implement routine varicella immunization for all children in 1995. Vaccination coverage in children 19-35 months of age reached 85% by 2003, and one-dose immunization was highly effective in reducing varicella-associated morbidity, ambulatory visits, hospitalisations and mortality, including indirect benefits observed in non-vaccinated groups [e.g., [14-17]. However, varicella outbreaks were still observed even in populations with high vaccination coverage; hence, the recommendations were expanded to a routine two-dose schedule in 2006 [18].

Germany was the first country in Europe to introduce funded nationwide varicella vaccination as part of the routine childhood vaccination schedule. In 2004, the German Advisory Committee on Vaccinations (STIKO) recommended vaccination with a single dose in children aged 11-14 months. Additionally, catch-up vaccinations for all susceptible children and adolescents were recommended, with two doses in children over 13 years of age [19]. Two monovalent varicella vaccines were available in Germany at the time of recommendation. A combined measles-mumps-rubella-varicella (MMR-V) vaccine, licensed with a two-dose schedule for all age groups, was available in 2006 [20]. In autumn 2008, licensure for both monovalent varicella vaccines was also changed to a two-dose schedule. In July 2009, the STIKO recommendation was adapted accordingly, with the second dose recommended preferably at 15-23 months of age [21].

Data on varicella vaccination coverage and the effects of the vaccination program is limited thus far in Germany. Based on extrapolation from statutory health insurance data varicella vaccination coverage for children 24 months of age in Germany was roughly estimated as 80% in 2009 [22]. Until March 2013, there was no statutory notification on varicella disease in Germany. From 2005 to 2009, nation-wide voluntary sentinel surveillance of about 900 paediatricians and general practitioners estimated a reduction in varicella cases by 63% in children <5 years of age, and by 55% in all age groups. This sentinel surveillance aimed at trends in varicella epidemiology, but could not provide estimates on varicella incidence as it was not population-based [20].

In October 2006 regional surveillance on children in a defined area (Munich) was implemented, the ‘Bavarian Varicella Surveillance Project’ (BaVariPro) [23]. In the first part of the project, annual cross-sectional parent surveys on random samples of children 18 to 36 months of age were performed from 2006 to 2011 to determine vaccination coverage in the area [24]. In the second part, routine varicella vaccination in paediatric practices and its impact on the frequency and age distribution of varicella cases was investigated from October 2006 to September 2011, to estimate incidences of varicella cases and to determine the frequency of varicella in vaccinated children, and of complicated varicella cases. In a third part of the project, database records from paediatric hospitals in the surveillance area were evaluated to identify varicella patients admitted during the years 2005 to 2009, and annual incidences for varicella-associated hospitalisations were calculated.

## Methods

### Surveillance area and study population

The area under surveillance was the City of Munich and its surrounding districts. The study population from 2005 to 2010 consisted of a yearly average of 237,700 children <17 years of age, with an increase in annual births from about 15,930 in 2005 to 17,370 in 2010. Approximately 77% of the paediatric population live in Munich City (Bavarian Statistical Office) [25]. At the start of surveillance, there were 132 registered paediatric practices and four paediatric hospitals in the area of Munich.

### Parent surveys

From 2006 to 2011, annual parent surveys were conducted at the end of each year to determine varicella vaccination coverage in children 18 to 36 months of age in the surveillance area, following the method described by Streng et al. [24]. For each survey, a random sample of 600 children aged 18-36 months was drawn from central inhabitant registries. The parents received a questionnaire by mail, including questions on history of varicella disease and the varicella and measles vaccination status as recorded in the child’s vaccination booklet (vaccine trade names and dates of vaccinations).

### Paediatric practice surveillance

In Germany, medical care and vaccination of pre-school children is mainly provided by paediatric practices. In September 2006, all 132 paediatric practices in the surveillance area were invited to participate in the regional monthly surveillance. Practices which no longer participated in January 2009 were replaced, aiming at a constant number of about 75 to 85 regularly reporting practices per month. At the start of surveillance, all participating practices provided a questionnaire on characteristics of the practice and the paediatrician. Practice surveillance was conducted from October 2006 to September 2011, thus including five completed varicella seasons (October to September).

Practices were asked to report all varicella patients according to the case definitions of the German ‘Working Group on Varicella’ [20]: a) children with a typical or atypical exanthema of the skin or mucous membranes compatible with varicella; b) children who had been varicella-vaccinated at any time before the start of varicella exanthema; c) children with varicella or its complications, who had to be hospitalized, or required antiviral or antibiotic therapy, or showed neurological symptoms, or showed involvement of organs other than the skin, or showed thrombocytopenia. Laboratory confirmation of varicella zoster virus was encouraged but not requested.

Varicella cases were reported aggregated by age group (<1, 1-4, 5-9, and 10-16 years of age) on monthly questionnaires. Additionally, the practices documented the monthly numbers of varicella and/or measles vaccinations (first and second dose), separately for monovalent varicella vaccine, measles-mumps-rubella (MMR) and combined MMR-varicella vaccine (MMR-V). Vaccinations were also documented stratified by age group (<2, 2-9, and 10-16 years of age). Finally, practices reported the total number of patient contacts during the respective month. In addition, pseudonymous, single-case questionnaires were collected for children with varicella complications, including basic demographic data, type of varicella complication, varicella vaccination status, chronic underlying diseases, hospitalisation, and outcome. The practices received a small compensation for each returned questionnaire. Missing, incomplete or implausible questionnaires were followed up by phone.

### Paediatric hospital database queries

All paediatric hospitals (n = 4) in Munich were invited to participate in database queries on patients <17 years of age with an ICD-10 (International Classification of Diseases, 10^th^ version) discharge diagnosis of varicella (B01.0 – B01.9) as primary or as any secondary diagnosis, admitted between January 1^st^, 2005 and December 31^st^, 2009. Basic demographic data (including age, gender, first three digits of postal code), date of hospitalisation, as well as the primary and all discharge diagnoses were collected. All reported varicella patients treated in Munich paediatric hospitals were included in the analysis of patient characteristics.

### Ethical consideration

The Bavarian Varicella Surveillance Project was approved by the Bavarian Data Protection Office and the University Ethics Committees of Munich and Wurzburg. For the parent surveys informed consent was required.

### Data analysis

Data from the parent surveys and practice surveillance were entered twice into a Microsoft Access 2003 database and reconciled. Data received from hospitals were entered into a Microsoft Excel 2003 database. All data were transferred into SPSS 20.0 or SAS 9.2 for statistical analysis. Data from the parent surveys and the hospitals were analysed descriptively per year, data from the practice surveillance per varicella season (October to September), providing percentages, mean and 95% confidence intervals (95%CI) or median with inter-quartile range (IQR). Comparisons across subgroups were assessed using Pearson’s Chi^2^-test for categorical data, or Mantel-Haenszel’s Chi^2^-test for linear trend, and the Kruskal-Wallis test for quantitative data using a 0.05 level of significance, respectively.

Univariate time series regression models were applied to analyse the age-specific time-course of varicella cases in the practices, following the statistical methods described by Höhle et al. [22]. Relative reduction factors (RRF) from season to season were estimated per age group from model-predicted values, with overall percentage of reduction across the seasons calculated as 1-(RRF_2/1_ x RRF_3/2_ x RRF_4/3_ x RRF_5/4_) for each age group [22].

For incidence estimates of varicella cases per season based on the paediatric practice surveillance the population at risk was assumed to be the resident paediatric population in Munich (City and surrounding districts) at 31^st^ of December [25]. Minimum incidences for each season and stratified by age group were calculated based solely on the number of observed cases in the participating practices. In a second approach, incidences were estimated taking into account varicella cases outside the scope of the present surveillance. Firstly, varicella cases in non-participating paediatric practices (36%-40%) were estimated, assuming a similar average number of varicella cases in a participating and a non-participating practice per season. Secondly, estimates on paediatric varicella cases treated by general practitioners (GP) were included. Based on a GP survey on about 10% of all registered GP practices in Munich (Streng et al.; unpublished data), it was estimated that, overall, the total number of more than 1,000 GP practices in the area contribute a total of 35% of all varicella cases <17 years of age for whom a physician was consulted. Thirdly, varicella cases not treated by a physician were estimated as (at least) 20% of all varicella cases per season, based on the results of the parent surveys on toddlers from the Munich area [24].

For annual incidence estimates on varicella-associated hospitalisations based on the data from Munich paediatric hospitals, the population at risk was assumed to be the resident paediatric population in Munich City at 31^st^ of December [25]. From all reported varicella patients, only children with a postal code specific for Munich City (803 to 819) were selected (as Munich District codes also cover some adjacent geographical areas). Furthermore, all patients with a primary diagnosis considered unrelated to varicella disease were excluded (for example, patients with overt surgical codes). For the years during which only three out of all four paediatric hospitals participated, the total annual number of paediatric varicella hospitalisations in Munich was estimated based on the number of hospital beds.

For those varicella seasons for which complete data from all paediatric hospitals were available, data from Munich City patients provided by the hospitals (age, sex, month of admittance, varicella complication diagnoses) were matched with single-case reports on patients reported as hospitalised by the paediatric practices, in order to evaluate the degree of underreporting of hospitalisations by the practice surveillance.

## Results

### Parent surveys - vaccination coverage

**Figure 1:**
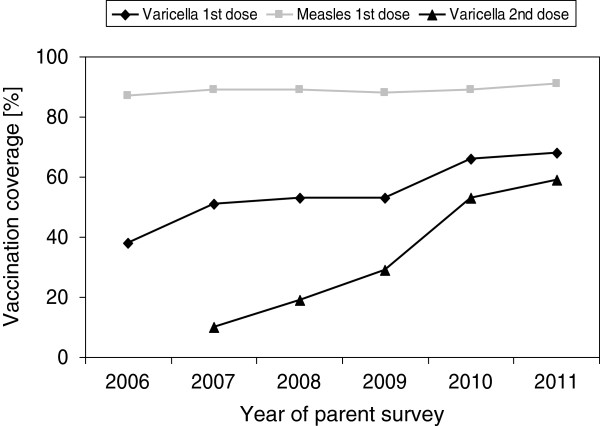
**Vaccination coverage for varicella (1**^**st **^**dose and 2**^**nd **^**dose) and for measles (1**^**st **^**dose) in children 18-36 months of age from annual parent surveys in the surveillance area (Munich), in percent.** In autumn 2008, licensure for all varicella vaccines available in Germany was changed to a two-dose schedule. Data from BaVariPro, parent surveys Nov 2006- Nov 2011.

For the surveys from 2006 to 2011, between 301 (50.2%) and 372 (62.0%) questionnaires were returned (Table 1). History of any previous varicella infection from 2006 to 2011 varied between a maximum of 18% (2007) and a minimum of 5% (2011; Table 1). During the period of recommended one-dose varicella vaccination, coverage of first-dose vaccinations was reported as 38% in 2006, 51% in 2007 and 53% in 2008 (see [24]). In July 2009, the second dose was recommended; 1^st^ dose coverage increased from 53% in 2009 to 66% in 2010 and 68% in 2011 (Table 1, Figure 1). For second-dose varicella vaccinations, recorded since 2007, the overall rates were below 20% until 2008; after recommendation of the two-dose schedule, second-dose vaccinations strongly increased (up to 59% in 2011; Table 1, Figure 1). In varicella-vaccinated children, use of solely tetravalent MMR-varicella vaccine was below 30% from 2006 to 2008 (2006: 2.2%, 2007: 19.0%, 2008: 28.7%), and increased strongly after 2008 (2009: 48.8%, 2010: 88.0%, 2011: 90.8%). During the total observation period from 2006 to 2011, vaccination coverage for measles (1^st^ dose) was between 86.9%, and 91.1%, respectively (p-value for linear trend =0.163; Figure 1).

**Table 1:** History of varicella and varicella vaccination coverage in children 18-36 months of age during five consecutive years; absolute numbers and percentages

**Parent survey on children 18-36 months of age**	**2006**	**2007**	**2008**	**2009**	**2010**	**2011**	p-value*
**(N questionnaires)**	**(N = 372)**	**(N = 364)**	**(N = 352)**	**(N = 330)**	**(N = 301)**	**(N = 302)**	
History of any previous varicella disease; n (%)	47 (12.6)	66 (18.1)	39 (11.1)	26 (7.9)	23 (7.6)	15 (5.0)	<0.001
Vaccinated against varicella at time of survey							
- received at least one dose; n (%)	140 (37.6)	187 (51.4)	188 (53.4)	174 (52.7)	200 (66.4)	206 (68.2)	<0.001
- received two doses; n (%)	N.A.	37 (10.2)	66 (18.8)	97 (29.4)	158 (52.5)	177 (58.6)	<0.001

Data from BaVariPro Parent Surveys; 2006 to 2011.

*Mantel-Haenszel’s Chi^2^-Test for linear trend.

### Practice surveillance – characteristics of participating practices and available reports

At the start of the practice surveillance in October 2006, 88 (67%) from 132 paediatric practices consented to participate. In total, 98 (74%) Munich practices provided practice questionnaires and monthly data during the surveillance period.

At the start of surveillance, participating paediatricians had practiced for an average of 21 years (median 20, range 8-53). On average, a practice consisted of two physicians (median 2, range 1-4) and three consulting-room assistants (median 3, range 0-7). Of the 98 practices, 22 (22%) treated up to 750 patients, 61 (62%) between 750 and 1,500 patients, and 15 (15%) more than 1,500 patients per month. During the quarter of the year prior to start of surveillance, the practices performed an average number of 30 routine well-baby visits (range 5-110) scheduled at an age of 10 to 12 months, and of 26 routine child visits (range 2-70) scheduled at an age of 5-6 years. On average, the practices had treated 23 varicella patients (range 0-82) during the quarter prior to start of surveillance, and had performed 23 vaccinations against varicella (range 0-60) and 72 (range 5-200) against measles.

A total of 4,914 monthly reports, covering 4,558,682 patient contacts, were received from all participating practices during the five seasons, with 82 reports per month on average (median 81, IQR 80-84, range 78-88 reports). From these reports, 3,600 (73%) were provided by 60 practices that reported data continuously during all 60 months of surveillance. The results from this subgroup were consistent with the results from the total sample.

For all cases with complications documented on the monthly reports, additional single-case reports were available (n = 134).

### Practice surveillance - vaccinations for varicella and measles

From October 2006 to September 2011, a total of 50,049 first-dose varicella vaccinations and of 40,541 second dose vaccinations were administered (Table 2). First-dose vaccinations increased from a monthly average of 9.5 per 1,000 patient contacts in 2006/07 to 14.0 until 2008/09 and decreased slightly to 12.9 in 2010/11. Second dose vaccinations were rare until 2007/08, with 1.0-2.2 per 1,000 patient contacts, increased to a monthly average of 15.1 per 1,000 patient contacts in 2008/09 after the change in licensure for the monovalent vaccines to a two-dose schedule in September 2008, and remained at 14.8-15.7 until 2010/11.

**Table 2:** Varicella vaccinations <17 years of age in Munich paediatric practices during five consecutive varicella seasons; absolute numbers and mean number per 1000 patient contacts (with 95% confidence interval, CI)

**Season**	**1**	**2**	**3**	**4**	**5**	**1-5**
**Surveillance period**	**Oct 06-Sep 07**	**Oct 07-Sep 08**	**Oct 08-Sep 09**	**Oct 09-Sep 10**	**Oct 10-Sep 11**	**Oct 06-Sep 11**
**(N all monthly reports)**	**(N = 1015)**	**(N = 965)**	**(N = 1013)**	**(N = 974)**	**(N = 947)**	**(N = 4914)**
Varicella vaccinations - all; 1^st^ dose						
- n	8234	9928	11872	10247	9768	**50049**
- mean per 1000 patient contacts (95%CI)	9.53 (9.03-10.03)	11.52 (10.96-12.09)	13.96 (13.30-14.63)	12.98 (12.32-13.65)	12.86 (12.25-13.48)	**12.17 (11.89-12.44)**
Varicella vaccinations - all; 2^nd^ dose						
- n	809	1703	13292	12967	11770	**40541**
- mean per 1000 patient contacts (95%CI)	0.98 (0.85-1.12)	2.17 (1.92-2.43)	15.12 (14.08-16.16)	15.68 (14.89-16.48)	14.76 (14.07-15.44)	**9.72 (9.36-10.07)**
Varicella vaccinations - monovalent; 1^st^ dose						
- n	6799	8289	3985	2095	1349	**22517**
- mean per 1000 patient contacts (95%CI)	7.73 (7.31-8.15)	9.43 (8.91-9.95)	4.32 (3.95-4.68)	2.71 (2.41-3.02)	1.93 (1.71-2.16)	**5.25 (5.06-5.44)**
Varicella vaccinations - monovalent; 2^nd^ dose						
- n	131	425	7524	5747	3553	**17380**
- mean per 1000 patient contacts (95%CI)	0.18 (0.12-0.24)	0.59 (0.42-0.75)	8.30 (7.52-9.08)	6.86 (6.35-7.37)	4.46 (4.13-4.78)	**4.09 (3.86-4.31)**
Varicella vaccinations - MMR-V; 1^st^ dose						
- n	1435	1639	7887	8152	8419	**27532**
- mean per 1000 patient contacts (95%CI)	1.80 (1.60-2.00)	2.10 (1.88-2.31)	9.66 (9.12-10.20)	10.30 (9.74-10.85)	10.95 (10.40-11.51)	**6.93 (6.70-7.16)**
Varicella vaccinations - MMR-V; 2^nd^ dose						
- n	678	1278	5768	7220	8217	**23161**
- mean per 1000 patient contacts (95%CI)	0.80 (0.69-0.92)	1.59 (1.41-1.76)	6.84 (5.90-7.31)	8.86 (8.36-9.37)	10.30 (9.77-10.83)	**5.64 (5.43-5.84)**

Data from BaVariPro Practice Surveillance; Oct 2006-Sep 2011.

**Figure 2:**
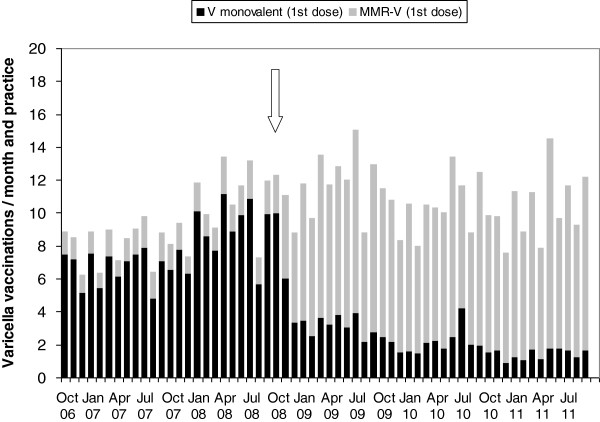
**Varicella vaccinations (1**^**st **^**dose), average per month and practice.** Stacked bars show proportion of monovalent varicella vaccine (black, n = 22,517) and of tetravalent MMR-V vaccine (grey, n = 27,532). The arrow indicates the change to a two-dose schedule for all licensed varicella vaccines. Data from BaVariPro, practice surveillance Oct 2006-Sep 2011.

**Figure 3:**
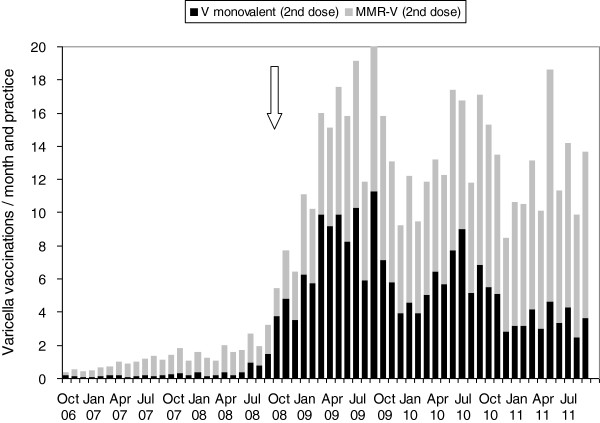
**Varicella vaccinations (2**^**nd **^**dose), average per month and practice.** Stacked bars show proportion of monovalent varicella vaccine (black, n = 17,380) and of tetravalent MMR-V vaccine (grey, n = 23,161). The arrow indicates the change to a two-dose schedule for all licensed varicella vaccines. Data from BaVariPro, practice surveillance Oct 2006- Sep 2011.

Until September 2008, monovalent varicella vaccine was used in 83% and MMR-V vaccine (reimbursed until then only in case of private health insurance) in 17% of all first-dose vaccinations (Figure 2). From October 2008 to September 2011, 66%-86% of first dose vaccinations were performed with MMR-V vaccine. For second dose vaccinations, both monovalent (43%) and MMR-V vaccine (57%) were regularly administered (Figure 3).

**Figure 4:**
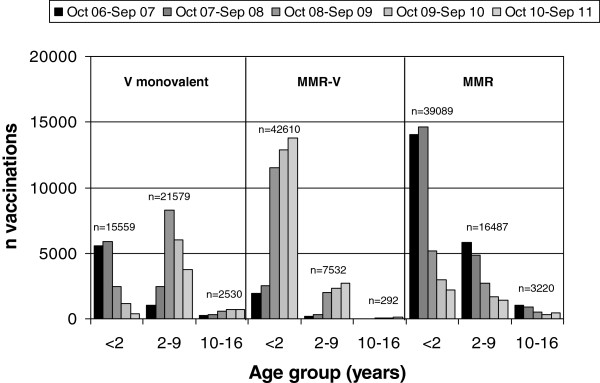
**Total number of vaccinations with monovalent varicella vaccine (V monovalent; n = 39,668), MMR-V vaccine (n = 50,434), and MMR vaccine (n = 58,796); per age group and season.** Data from BaVariPro, practice surveillance Oct 2006-Sep 2011.

The total number of all vaccinations (first and second dose) with monovalent and with MMR-V vaccine differed between age groups and seasons (Figure 4). In children <2 years of age, a total of 15,559 doses of monovalent varicella vaccine were administered, with a strong decrease during the observation period. In children 2-9 years of age, monovalent vaccine doses (n = 21,579) peaked during the 3^rd^ season 2008/09, and in children 10-16 years of age, monovalent vaccine doses (n = 2,530) increased over the seasons. MMR-V doses were mainly administered in children <2 years of age (n = 42,610), with a strong increase after September 2008. This increase was also observed in children 2-9 years of age, with 7,532 MMR-V doses in total, including children who received MMR-V as the second vaccination for measles and as the first vaccination for varicella. Corresponding to the increase of MMR-V doses, MMR doses in children <2 years of age (n = 39,089 doses) and in children 2-9 years of age (n = 16,487 doses) decreased after September 2008.

### Practice surveillance - varicella cases and incidence estimates

**Figure 5:**
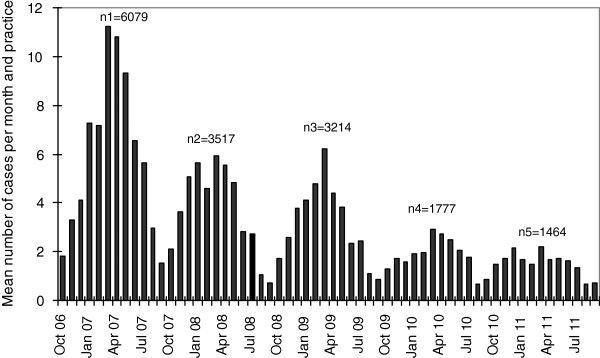
**Mean number of varicella cases per month and paediatric practice during five varicella seasons in the surveillance area (Munich), with total number of cases per season (Oct-Sep).** Data from BaVariPro, practice surveillance Oct 2006-Sep 2011.

**Figure 6:**
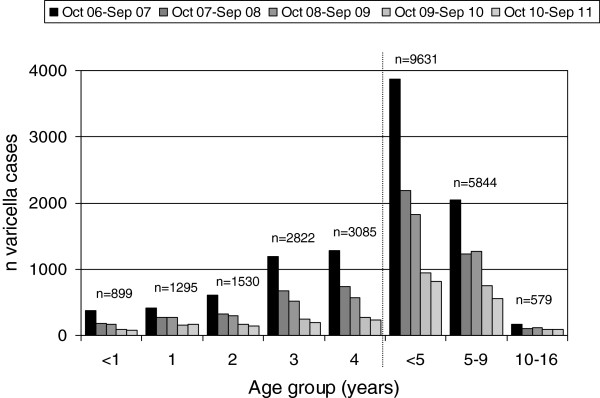
**Varicella cases (n = 16,054) per age group, during 5 varicella seasons (Oct 06-Sep 07, n1 = 6079; Oct 07-Sep 08, n2 = 3518; Oct 08-Sep 09, n3 = 3215; Oct 09-Sep 10, n4 = 1778; Oct 10-Sep 11, n5 = 1464).** Data from BaVariPro, practice surveillance Oct 2006-Sep 2011.

**Figure 7:**
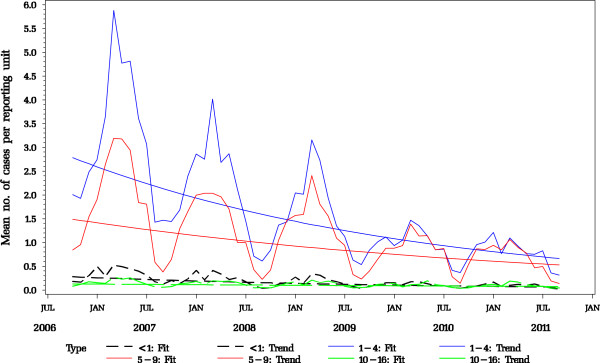
Model-fitted mean number of varicella cases per reporting unit (paediatric practice) by age group (<1, 1-4, 5-9, 10-16 years of age), with linear trends per age group from five varicella seasons (n tot. =16,054). Data from BaVariPro, practice surveillance Oct 2006 – Sep 2011).

From October 2006 to September 2011, the paediatricians reported a total of 16,054 varicella cases showing a typical annual pattern with peaks in spring of each year (Table 3, Figure 5). Varicella cases decreased from 6,079 cases (a mean of 6.6 cases per 1,000 patient contacts) in 2006/07 to 1,464 cases (2.2 cases per 1,000 patient contacts) in 2010/11, thus showing an overall decrease in the mean number of cases by 67% during the five seasons (Figure 5). The decrease occurred in two steps, and was strongest between the first and second season (by 2.4 cases per 1,000 patient contacts). The number of varicella cases reported in the seasons 2 and 3 were similar (decrease of 0.25 per 1,000 patient contacts), followed by a further pronounced decrease in season 4 (of 1.7 cases per 1,000 patient contacts), whereas season 5 was similar again to season 4 (decrease of only 0.1 cases per 1,000 patient contacts). The majority of cases were observed in children <5 years of age (Figure 6). The mean number of cases and their linear trends for the age groups <1, 1-4, 5-9 and 10-16 years are shown in Figure 7. During the five years of surveillance, overall age-related reductions of 71% in children <1 year of age, 75% in 1-4 year old children, 63% in 5-9 year-old children, and 63% in 10-16 year old children were observed (for the magnitude of changes in the different age groups from each season to the next, see Table 4).

**Table 3:** Varicella cases in children <17 years of age in Munich paediatric practices during five consecutive varicella seasons; absolute numbers and mean number per 1000 patient contacts (with 95% confidence interval)

**Season**	**1**	**2**	**3**	**4**	**5**	**1-5**
**Surveillance period**	**Oct 06-Sep 07**	**Oct 07-Sep 08**	**Oct 08-Sep 09**	**Oct 09-Sep 10**	**Oct 10-Sep 11**	**Oct 06-Sep 11**
**(N all monthly reports)**	**(N = 1015)**	**(N = 965)**	**(N = 1013)**	**(N = 974)**	**(N = 947)**	**(N = 4914)**
Varicella cases - all						
- n	6079	3518	3215	1778	1464	**16054**
- mean per 1000 patient contacts (95%CI)	6.58 (6.14-7.03)	4.20 (3.85-4.55)	3.95 (3.59-4.30)	2.26 (2.02-2.51)	2.16 (1.86-2.47)	**3.86 (3.70-4.02)**
Varicella cases - vaccinated						
- n	218	231	314	179	134	**1076**
- mean per 1000 patient contacts (95%CI)	0.27 (0.22-0.32)	0.32 (0.24-0.40)	0.40 (0.34-0.47)	0.26 (0.21-0.32)	0.24 (0.17-0.32)	**0.30 (0.27-0.33)**
- % of all varicella cases	3.6	6.6	9.8	10.1	9.2	**6.7**
Varicella cases - with complications						
- n	31	20	38	28	17	**134**
- mean per 1000 patient contacts (95%CI)	0.04 (0.02-0.06)	0.03 (0.01-0.05)	0.07 (0.03-0.11)	0.07 (0.02-0.11)	0.05 (0.01-0.08)	**0.05 (0.04-0.07)**
- % of all varicella cases	0.5	0.6	1.2	1.6	1.2	**0.8**

Data from BaVariPro Practice Surveillance; Oct 2006-Sep 2011.

**Table 4:** Relative reduction factors (RRF) for varicella cases per age group (model-predicted, see Höhle et al. 2010)

**Ratio**	**RRF**	**RRF**	**RRF**	**RRF**
	**Season 2 / Season 1**	**Season 3 / Season 2**	**Season 4 / Season 3**	**Season 5 / Season 4**
**Age group (years)**				
<1	0.698	0.743	0.670	0.815
1-4	0.676	0.749	0.594	0.847
5-9	0.732	0.906	0.660	0.842
10-16	0.856	0.908	0.868	0.927

Data from BaVariPro Practice Surveillance, Oct 2006-Sep 2011.

Minimum incidence estimates per 1,000 children <17 years of age, based on the number of reported cases, decreased by 76.9%, from 26.0 per 1,000 children <17 years of age in the first season to 6.0 in the fifth season (Table 5); for the age groups <1, 1-4, 5-9, and 10-16 years, the incidences decreased by 79.9%, 80.6%, 73.9% and 47.4%, respectively. Taking into account paediatric varicella cases occurring in paediatric and general practitioner practices outside the surveillance project, as well as children not seeking medical care, varicella incidences were estimated approximately three times as high (Table 5), with similar decreases assuming similar vaccination coverage.

**Table 5:** Seasonal incidence estimates of varicella cases per 1000 children 0-16 years of age in the area of Munich, by age group; a) minimum incidence estimate based on observed cases in paediatric practices, b) incidence estimate taking into account estimates on varicella cases from non-participating paediatric practices, general practitioners, and cases without physician consultation

**Season**	**1**	**2**	**3**	**4**	**5**	**1-5**
**Surveillance period**	**Oct 06-Sep 07**	**Oct 07-Sep 08**	**Oct 08-Sep 09**	**Oct 09-Sep 10**	**Oct 10-Sep 11**	**Oct 06-Sep 11**
a) Incidence /1000; based on observed numbers of cases in the participating practices (minimum incidence)						
<1 year of age						
- n	372	182	168	96	81	**899**
- incidence	23.4	10.9	9.8	5.6	4.7	**10.7**
1-4 years of age						
- n	3494	2004	1652	845	737	**8732**
- incidence	58.7	33.2	26.7	13.3	11.4	**28.2**
5-9 years of age						
- n	2039	1226	1273	752	554	**5844**
- incidence	29.9	18.0	18.6	10.9	7.8	**16.9**
10-16 years of age						
- n	174	106	122	85	92	**579**
- incidence	1.9	1.2	1.3	0.9	1.0	**1.3**
<17 years of age (all cases)						
- n	6079	3518	3215	1778	1464	**16054**
- incidence	26.0	14.9	13.5	7.4	6.0	**13.4**
b) Incidence /1000; corrected for cases from non-participating practices in the respective season^*^, cases from general practitioner practices^#^, and untreated cases^§^ (estimates)						
<17 years of age (all cases)						
- n	18244	11106	9667	5561	4710	**49288**
- incidence	78.1	47.1	40.6	23.0	19.2	**41.3**

Data from BaVariPro Practice Surveillance, Oct 2006-Sep 2011.

*: assuming that, on average, a participating and a non-participating paediatric practice contribute similar numbers of varicella cases in each season.

#: assuming that cases from general practitioner practices represent approximately 35% of all varicella cases treated by a physician.

§: assuming that, on average, untreated cases represent approximately 20% of all paediatric varicella cases in each season (Streng et al. [24]).

A total of 1,076 (6.7%) varicella cases in vaccinated children were documented, following the seasonal pattern of varicella disease. A maximum of 314 cases (0.4 per 1,000 patient contacts) was reported in season 3 and a minimum of 134 cases (0.2 per 1,000 patient contacts) in season 5 (Table 3). Absolute numbers of vaccinated cases were lowest in season 4 and 5.

Complications of varicella occurred in 134 (0.8%) practice patients (Table 3) and also peaked in spring of each year. During all five seasons, complicated cases were observed in the range of 0.03 to 0.07 cases per 1,000 patient contacts (Table 3). From all 134 patients, 86 (64.2%) were <5 years of age, 46 (34.3%) were 5 to 9 years old, and 2 (1.5%) 10 to 16 years old; across all five seasons, complications were most frequent in children <5 years of age. For 11 (8.2%) patients with complications, an underlying chronic disease was documented. For all 134 patients, a total of 190 specific complications were reported (55/25/45/39/26 in seasons 1 to 5); most frequent were infectious skin complications (n = 66) and otitis media (n = 45). Complications of the central nervous system were observed in nine children (3/1/2/2/1 in seasons 1 to 5), along with encephalitis (n = 2), meningitis (n = 2), febrile convulsions (n = 2), cerebellitis (n = 1), status epilepticus (n = 1), and strong headaches (n = 1). Seventeen (12.7%) from 134 children with complications were breakthrough varicella cases >42 days after one-dose vaccination. This corresponds to a complication rate of 0.1% in vaccinated children, compared to 0.7% in unvaccinated children. From a total of nine patients with complications of the central nervous system, two (22%) occurred in children with breakthrough varicella after a single dose (febrile convulsions).

Hospitalisation was reported for 23 practice patients (8/4/4/3/4 in seasons 1 to 5), corresponding to 0.1% of all 16,054 patients captured by the practice surveillance and 17% of all 134 patients with complications. Five of the hospitalized children were breakthrough varicella cases at least 7 months after vaccination, including two children with underlying chronic disease (one child with neurodermitis had a complicated febrile seizure, the other was a child with pre-existing HIV infection hospitalized for acyclovir therapy). No fatalities occurred; for five (3.7%) of all 134 children (0.03% of all 16,054 children) permanent scars were documented.

### Hospital databases – patient characteristics and hospitalisation incidence

From 2005 to 2009, a total of 178 varicella patients were reported from the hospitals. For the years 2005 and 2006 three out of all four Munich paediatric hospitals, covering 88% of all paediatric hospital beds in the area, provided data (28 patients in 2005, 34 in 2006); for the years 2007 to 2009, data were available from all four hospitals (62 in 2007, 31 in 2008, 23 in 2009).

The majority (116; 65.2%) were <5 years of age (including 38 children <11 months of age), 47 (26.4%) were 5 to 9 years old, and 15 (8.4%) were 10 to 16 years of age; 90 (50.6%) were male children. In 162 (92.3%) children the primary diagnosis either was varicella (82; 46.1%) or a known complication of varicella disease (70; 39.3%) or indicated that the child belonged to a risk group for severe varicella (10; 5.6%). A total of 21 (11.8%) patients suffered from an immunocompromising condition. Complications of the central nervous system were reported in 40 (22.5%) patients, including febrile convulsions (12), other convulsions (7), encephalitis (7), syncope / collapse (3), and facial paralysis (3) as the most frequent neurological complications. Fifty-seven (32.0%) patients had gastrointestinal complications (mainly difficulties to feed or drink / dehydration; n = 32). Also frequent were complications of the skin in 28 (15.7%) patients (mainly local skin infections; n = 18), and of the lower respiratory tract in 16 (9%) patients, including eight children with varicella-associated pneumonia. Median duration of hospital stay was 3 days (IQR 2-6). One fatality was reported in an 8-months-old girl with varicella pneumonia suffering from pre-existing chronic conditions (severe perinatal asphyxia, microcephaly / hydrocephaly, cardiac defect), admitted in July 2009 with acute respiratory distress syndrome; death occurred after one day in the hospital.

Out of all 178 varicella patients treated in Munich paediatric hospitals, 113 (63.5%) had a postal code compatible with the complete surveillance area Munich City and its surrounding districts; 105 of these were varicella-related hospitalisations, including 71 with a specific postal code for Munich City. With an estimate of two additional patients each in 2005 and 2006 for the non-participating hospital, incidence calculation of varicella-associated hospitalisations was based on a total number of 75 patients. From 2005 to 2008, incidence per 100,000 children <17 years of age varied between 7.6 and 12.1, and decreased to 4.3 in 2009 (by 43% since 2005; Table 6). For the age group <5 years of age, hospitalisation incidences were between 15.2 and 21.2 until 2008 and decreased to 4.7 in 2009 (by 78% since 2005; Table 6). The strongest decrease was observed in children <1 year of age, from a maximum incidence of 60.8 in 2005 to a minimum of 14.2 in 2009 (by 77% since 2005; Table 6).

**Table 6:** Annual incidences of varicella-associated hospitalisations per 100,000 children (Munich City) 0-16 years of age, from Munich paediatric hospitals, by age group

**Year of hospitalisation**	**2005**	**2006**	**2007**	**2008**	**2009**	**2005-2009**
<1 year of age						
- n	8	3	5	4	2	**22**
- incidence /100,000	60.8	26.2	36.5	28.6	14.2	**32.5**
<5 years of age						
- n	12	9	13	12	3	**49**
- incidence /100,000	21.0	15.2	21.2	19.0	4.7	**15.9**
<17 years of age (all cases)						
- n	14	17	22	14	8	**75**
- incidence /100,000	7.6	9.4	12.1	7.6	4.3	**8.2**

Data from BaVariPro Hospital Databases, 2005-2009.

### Matching of hospital and paediatric practice patients

For season 2 and 3 from the practice surveillance (Oct 2007 to Sep 2009), a total of 23 varicella patients (16 and 7 respectively) from Munich City were reported from all four hospitals. For season 2, 12 (75%) out of 16 hospital patients had either not contacted a paediatrician or were patients from practices not covered by the surveillance. For season 3, six (86%) out of seven hospital patients had not been captured by the practice surveillance.

## Discussion

This is the first study in Germany and Central Europe providing incidence estimates for changing varicella epidemiology after recommendation of routine vaccination. In a defined surveillance area, data on children collected from parents, paediatric practices and hospitals indicated increasing acceptance of vaccination and a clear decrease of the burden of varicella infections and complications.

After the recommendation in 2004, coverage in children up to three years of age in the surveillance area increased markedly (in two steps) during the observation period. During the one-dose vaccination schedule recommended until June 2009, varicella vaccination coverage at first increased to 51% in 2007 but then stagnated at 53% in 2008 [24] and 2009. During that first period, statutory reimbursement regulations in the surveillance area only covered the use of monovalent varicella vaccines (applicable in one dose). Combined MMR-varicella vaccine (applicable in two doses) was reimbursed by private health insurance only and was used in less than 20% of first-dose varicella vaccinations. After the recommendation of a two-dose schedule in 2009 and the resultant reimbursement also for the two-dose MMR-varicella vaccine, the combined vaccine was strongly preferred. In the last observed season of practice surveillance (Oct 2010-Sep 2011) more than 85% of all first-dose dose varicella vaccinations were performed with combined vaccine, and in the last parent survey (Nov 2011), more than 90% of the vaccinated children had received it. This is very likely due to the fact that vaccinations against MMR are generally well accepted, and that by the use of combination vaccines additional injections can be avoided [6,26-28]. Presumably due to this better acceptance, a second pronounced increase in first-dose varicella vaccination coverage up to 66% was observed in 2010. However, coverage stagnated again, at 68% in 2011. This is considerably below the advisable coverage level of 85%-90% to avoid a potential shift in the age-related incidence towards older children and adults [29]. In contrast, vaccination against measles was consistently high during the observation period (first dose coverage 86%-91%), indicating that a considerable proportion of parents (23% in 2011) accepted vaccination against measles but neither separate nor combined vaccination against varicella.

The two pronounced increases in coverage were each followed by two marked decreases in the number of paediatric varicella cases, from 6.6 to 4.2 cases per 1,000 patient contacts (season 1 to 2) and from 4.0 to 2.3 cases (season 3 to 4), with an overall decrease of 67% of all cases during the five-year observation. The strongest decrease (by 75%) was observed in children below five years of age, i.e. the age group most frequently affected by varicella disease in unvaccinated populations [1]. Children below one year of age, 80% of whom are not yet varicella-vaccinated [24], and older children and adolescents also showed a considerable decrease (71% and 63%, respectively), indicating herd protection effects. The overall decrease in incidence was 77% for all children.

In the participating practices, covering about two-thirds of all paediatric practices in the area, varicella complications were rarely observed (<1% of all varicella cases), and the practices reported only 23 hospitalisations during the five-year observation period. However, the practice surveillance only captured 14% to 25% of all varicella cases treated in the paediatric hospitals in the surveillance area, indicating that a considerable proportion of complicated cases presented directly at the hospitals without the paediatrician being aware of this fact. Thus, the true burden of varicella complications and hospitalisations may be underestimated by surveillance systems based on practices only. Our data confirm the strong underreporting of hospitalisations already suspected for the German sentinel surveillance, where about 900 practices reported only 71 hospitalisations during four years of observation [30]. The data collected directly from the hospitals in our surveillance area showed a strong impact of vaccination on the incidence of varicella-related hospitalisations, which decreased by 43% during five years. It should be noted that the 5 years of hospital surveillance started earlier than the other parts of the project and the last data were collected in 2009. Thus, the hospital data were collected largely during the one-dose vaccination schedule; nevertheless, considerable reductions of these more severe cases were already observed (by 78% in children <5 years of age). This confirms results from other studies indicating the high effectiveness of varicella vaccination for prevention of severe cases [e.g., [15]. The only varicella-associated fatality observed during the study occurred in an 8-months-old child with severe underlying chronic disease, thus indicating the importance to vaccinate close contacts of persons at high risk for serious varicella complications.

Vaccinated varicella cases were rare (on average, 7% of all cases) and, after initial increase, remained stable at a proportion of 9%-10% after season 3. At the end of season 3, the second dose had been recommended. Vaccinated cases after two doses usually occur less frequently than after a single dose [31]. The reduction observed after season 3 may be either due to the boosting effect of the second dose [32] during that time, or a result of a general increase in varicella vaccination coverage in the population. Due to the high effectiveness of varicella vaccines and the resultant decrease of unvaccinated cases, the proportion of vaccinated cases is expected to further increase over time [18].

Compared to German regions with earlier reimbursement of one- and two-dose varicella vaccinations, and hence presumably higher coverage, the impact of the vaccination programme was delayed in our surveillance region [20,23]. During four years of observation (2005/2006 until 2008/2009) and including all age groups, German sentinel surveillance found a largely continuous reduction of annual varicella cases, with a total decrease by 63% of all cases [30], which preceded the similar decrease observed in our population by about two years. The difference is most likely due to the delay in reimbursement for vaccination in Bavaria, and demonstrates that besides the recommendations which were equal throughout Germany, reimbursement of vaccine is crucial to obtain the necessary coverage for control of varicella.

From Europe, a study from the Veneto region (Italy), investigated the effectiveness of two-dose varicella vaccination introduced in 2005, with the first dose in the second year of life and a second dose at the age of six years [33]. In this region, vaccination was well accepted and 79% first-dose coverage was reached in the 2008 birth cohort, with MMR-varicella vaccine being used in up to 92% of all vaccinations. Up to 64% decrease of paediatric varicella incidence and 53% of varicella hospitalisations were observed there until 2008 [33]. Further impact data are available from the Sicily region, where universal vaccination was introduced in 2003, with a coverage >85% and a decrease in paediatric incidence by >90% to 2007 [34]. A rapid and steep reduction in the incidence (by up to 96%) as well as in hospital admissions (by 71%) was also observed in the Navarre region (Spain) to 2010, where universal vaccination was introduced in 2007 [35]. Although seroprevalence studies indicated a lower age at varicella infection in Germany compared to Italy and Spain [34,36], and vaccination programmes differed in time and pattern of introduction, age at vaccination, acceptance and surveillance systems, routine varicella vaccination appears highly effective in all European regions investigated so far.

Apart from Europe and the USA, similarly high impact of publicly funded, mainly single-dose, routine varicella vaccination programmes in early childhood was reported in Uruguay [7] and more recently in Taiwan [37], Australia [38,39], Canada [40] and Costa Rica [41] (Table 7). In all these countries, higher levels of coverage were reached earlier, and stronger reductions found in a shorter time than in both German surveillance systems, reflecting a more reluctant attitude towards varicella vaccination by physicians and parents in Germany [24].

**Table 7:** Review on the impact of nation-wide, publicly funded, mainly one-dose, routine varicella vaccination programmes in early childhood in countries outside Europe and the US

**Authors**	**Year of publication**	**Country**	**Nationwide routine varicella vaccination implemented (year)**	**Coverage**	**Decrease in varicella patients reached (year)**
Quian et al. [7]	2008	Uruguay	1999	>90% in 2005	Outpatient visits by 87%, hospitalisations by 81% (2005)
Chao et al. [37]	2012	Taiwan	2004	>90% after 2006	Incidence by 53% (2008)
Carville et al. [38],	2010	Australia	2005	82% in 2009	Hospitalisation rates / community consultations by 7% (2007), congenital varicella by 100%, neonatal varicella by >85% (2008/2009)
Khandaker et al. [39]	2011				
Tan et al. [40]	2012	Canada	2007	58%-90% in 2008	Hospitalisations by 81%-86% (2008)
Ulloa-Gutierrez et al. [41]	2011	Costa Rica	2007	n.a.	Paediatric cases by 83% (2010)

A potential limitation of our practice surveillance study is that although the majority of all paediatric practices in the area participated, these practices may not be fully representative of the population investigated. Practices with a more negative attitude towards varicella vaccination may not have participated in the surveillance, and, hence, in those practices the average number of varicella cases may have been higher, potentially resulting in an underestimation of varicella incidence. Paediatricians and parents in the surveillance area may differ in their attitude towards varicella vaccination from other German regions, for which a higher coverage had been estimated [20,22]. The practice surveillance was based on observation of clinical varicella cases without laboratory confirmation; hence, mild cases in vaccinated children may have been underreported. Varicella hospitalisations assessed by ICD-10 based data from hospitals were not confirmed by chart review. However, misclassification seems to be unlikely due to the typical clinical diagnosis of varicella.

The WHO recommended in 1998: “Routine childhood immunization against varicella may be considered in countries where this disease is a relatively important public health and socioeconomic problem, where the vaccine is affordable, and where high (85%-90%) and sustained vaccine coverage can be achieved” [29]. In the USA, a coverage level of 85% was reached in the year 2003, seven years after the initial recommendation of a one-dose schedule. This may be partly due to implementation of day-care and school entry requirements for varicella vaccination [42]. In Germany and other European countries without such requirements, high coverage levels may be more difficult to achieve, and increased use of combined MMR-V vaccine might help to reach this goal. However, data on both the MMR-V vaccine used in the USA (Proquad®, manufactured by Merck, Whitehouse Station, NJ, USA) and the one used in Germany (Priorix Tetra®, manufactured by GlaxoSmithKline, Wavre, Belgium) indicated a slightly increased risk of febrile seizures at a period of 5 to 12 days after receipt of the first dose (estimated as approximately one additional febrile seizure per 2000 first-dose MMR-V vaccinations) compared to simultaneous but separate first-dose vaccination with MMR and monovalent varicella vaccine. Hence, the Advisory Committee on Immunization Practices in the USA recommended separate application of MMR and varicella vaccine for the first dose in 2010 [43]; the German STIKO gave a similar recommendation in September 2011 [44]. As observed in Munich during the previous period of vaccination mainly with monovalent varicella vaccines, it could be expected that some parents might again not accept this additional injection. Indeed, recent data from our surveillance project showed that first-dose varicella vaccinations decreased by 12% in the Munich paediatric practices during the year following the recommendation in 2011, whereas first-dose MMR vaccinations remained on the same level as the previous MMRV vaccinations (Streng et al., unpublished). This may contribute to stagnating varicella vaccine coverage and, over time, result in an increasing number of children with missing or incomplete protection at least in some regions of Germany. The potential consequences of such a vaccination pattern on varicella zoster virus epidemiology in Germany - such as disease resurgence or a shift to older age at infection - will have to be monitored closely. Long-term monitoring, as it has been suggested by a recent evaluation of the German varicella vaccination program [45], will be facilitated, as varicella was made a notifiable disease in Germany in March 2013 [46].

## Conclusions

After introduction of routine varicella vaccination in 2004, coverage increased up to 68% until 2011 in the surveillance area. Correspondingly, paediatric varicella cases decreased by 67% and paediatric hospitalisations by 43%. Preferential use of combined MMR-varicella vaccine since 2009 was linked to a marked increase in coverage. As MMR-V is currently not recommended for first-dose varicella vaccination, it may be difficult to maintain the present coverage levels or achieve higher coverage necessary for sustained control of varicella. Continuous surveillance of the varicella epidemiology and continuous evaluation of the current recommendations is needed to monitor the long-term effectiveness of varicella vaccination.

## Competing interests

AS and JGL received an unrestricted research grant for the present study from GlaxoSmithKline (GSK) Vaccines (Rixensart, Belgium) and research grants for other epidemiological studies. AS received honoraria from Sanofi Pasteur MSD for participating at an expert meeting and from GSK for study presentations during investigator meetings, as well as financial support for conference attendance from GSK. VG received lecturing fees and financial support for conference attendance from GSK. JGL received research grants for vaccine studies and further epidemiological studies as well as honoraria for participating at advisory boards and lecturing at medical conferences from both manufactures of varicella vaccines, GSK and Sanofi Pasteur MSD.

## Authors’ contributions

AS, VG and JGL designed and supervised the study. AS, DC, CH and JGL analysed and interpreted the data, and AS drafted the manuscript. All authors assisted in manuscript revision and approved the final manuscript version for publication.

## Pre-publication history

The pre-publication history for this paper can be accessed here:

http://www.biomedcentral.com/1471-2334/13/303/prepub
